# Supplementary material for: AlphaFold 3 accurately models natural variants of Helicobacter pylori catalase KatA
Source: bioRxiv. 2025 Jun 3:2025.06.02.657526. Preprint. [Version 1] doi: 10.1101/2025.06.02.657526 (PMC12157475; doi:10.1101/2025.06.02.657526)
Supplement: 2 [file NIHPP2025.06.02.657526v1-supplement-2.pdf]

bioRxiv preprint doi: https://doi.org/10.1101/2025.06.02.657526; this version posted June 3, 2025. The copyright holder for this preprint (which was not certified by peer review) is the author/funder, who has granted bioRxiv a license to display the preprint in perpetuity. It is made available under aCC-BY 4.0 International license.

Supplementary material

Table S1. Summary of crystallographic statistics.

Protein, PDB code	*H. pylori* KatA$_{SS1}$, PDB: 9NH3
Space Group	P 2_1 2 2_1
Cell dimensions and angle	67.5, 96.2, 154.8, 90, 90, 90
(a, b, c, α, β, γ) (Å, º)	
Resolution (Å) [a]	55.3-1.87 (1.94-1.87)
Completeness (%) [a]	100.0 (100.0)
Total reflections	1087793 (107235)
Unique reflections	84023 (8292)
Average *I/σ* [a]	8.45 (1.50)
R_{merge} [a]	0.28 (1.42)
$CC_{1/2}$ [a]	0.99 (0.57)
R_{work} (%)	14.3
R_{free} (%)	19.0
Ramachandran favored, allowed, outliers (%)	96.0, 4.0, 0.0
Non-hydrogen atoms	9435
Solvent atoms	1205
Protein chains, residues	2, 978
Average B-factor of protein atoms ($Å^2$)	17
Average B-factor of solvent atoms ($Å^2$)	26
rms bond lengths (Å)	0.010

bioRxiv preprint doi: https://doi.org/10.1101/2025.06.02.657526; this version posted June 3, 2025. The copyright holder for this preprint (which was not certified by peer review) is the author/funder, who has granted bioRxiv a license to display the preprint in perpetuity. It is made available under aCC-BY 4.0 International license.

rms bond angles (°)	1.00
TLS groups	17
Molprobity clash score, percentile	2.24, 99th

[a] Values in parentheses indicate statistics for the highest resolution shell.

Data S1. Sequences of *H. pylori* KatA variants.
